# Supplementary material for: Myeloperoxidase Alters Lung Cancer Cell Function to Benefit Their Survival
Source: Antioxidants (Basel). 2023 Aug 9;12(8):1587. doi: 10.3390/antiox12081587 (PMC10451743; doi:10.3390/antiox12081587)
Supplement: Supplementary file 1 [file antioxidants-12-01587-s001.zip › antioxidants-2417406-supplementary.pdf]

Myeloperoxidase Alters Lung Cancer Cell Function to Benefit Their Survival

Nejra Cosic-Mujkanovic [1,†], Paulina Valadez-Cosmes [1,†], Kathrin Maitz [1], Anna Lueger [1], Zala N. Mihalic [1], Marah C. Runtsch [1], Melanie Kienzl [1,2], Michael J. Davies [3], Christine Y. Chuang [3], Akos Heinemann [1,2], Rudolf Schicho [1,2], Gunther Marsche [1,2], and Julia Kargl [1,2,*]

[1] Division of Pharmacology, Otto Loewi Research Center, Medical University of Graz, 8010 Graz, Austria
[2] BioTechMed-Graz, 8010 Graz, Austria
[3] Department of Biomedical Sciences, Panum Institute, University of Copenhagen, DK-2200 Copenhagen, Denmark
* Correspondence: julia.kargl@medunigraz.at
† These authors contributed equally to this work.

Supplementary Data:

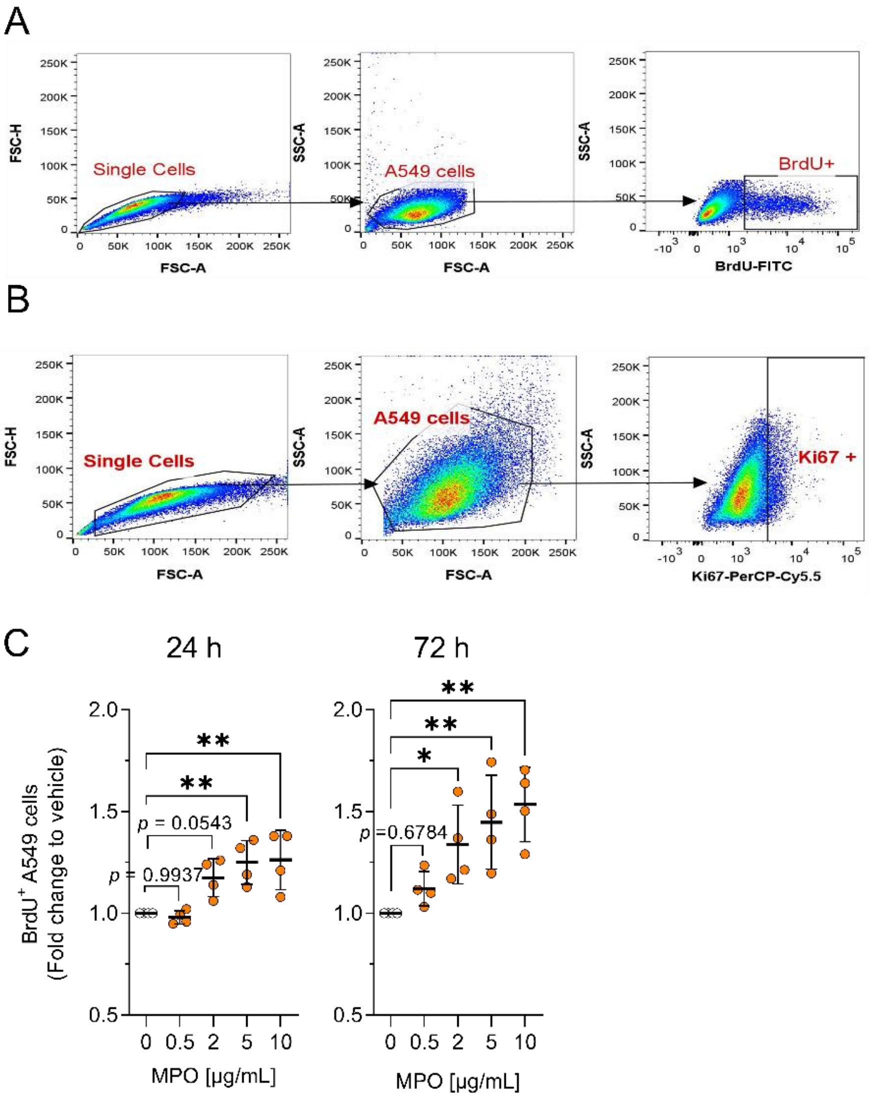

Figure S1: A549 cell proliferation. Representative scatter plots of flow cytometry gating strategies used to analyse (**A**) BrdU and (**B**) Ki67 stained cells. (**C**) MPO effects on proliferation after 24 and 72 h of MPO treatment assessed with the BrdU assay. Data indicate mean values ± SD from 4 independent experiments. Statistical differences were assessed using one-way ANOVA with Dunnett´s post hoc test. * $p < 0.05$, ** $p < 0.01$.

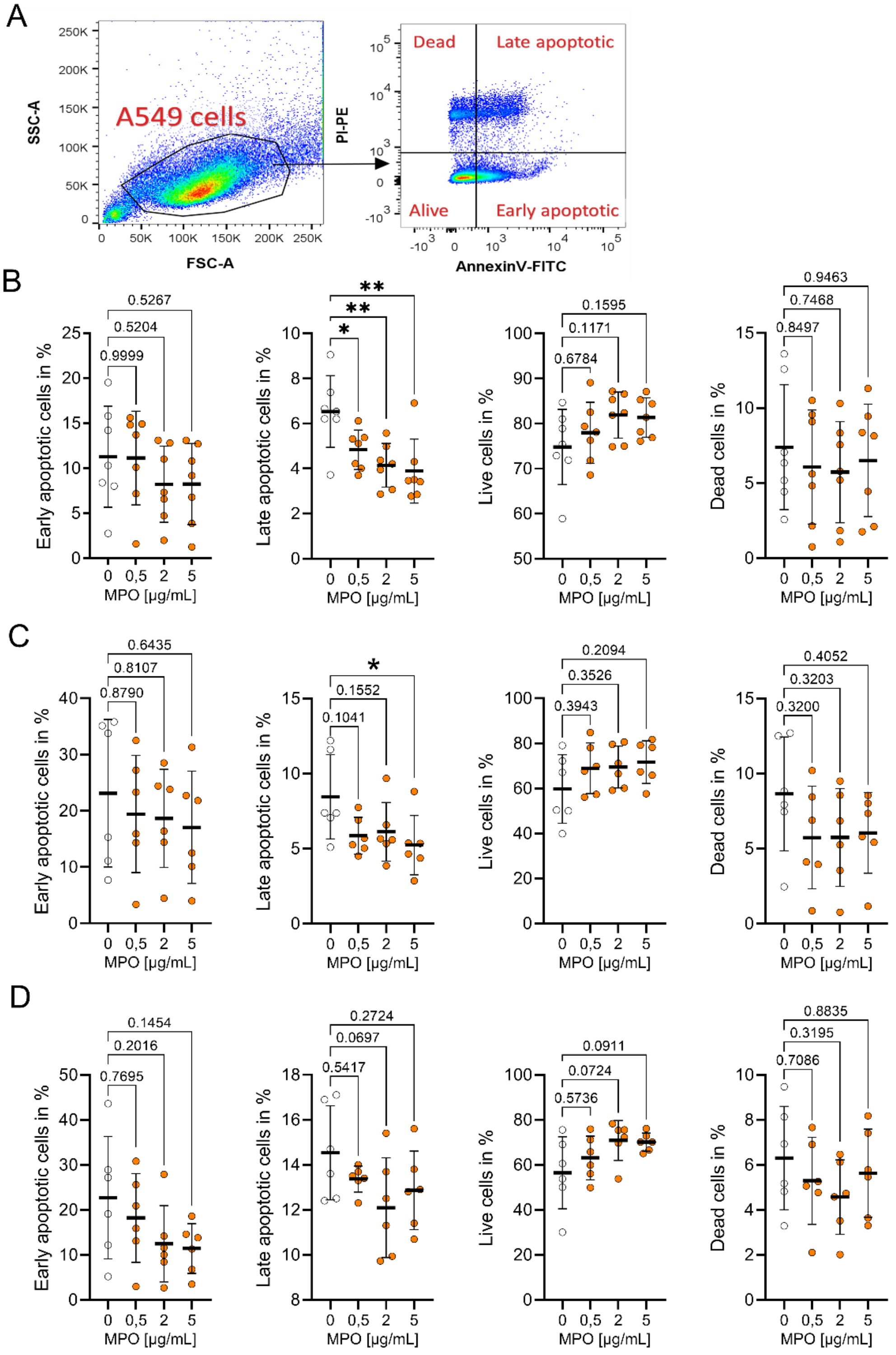

Figure S2: Apoptosis of A549 cells. **(A)** Representative scatter plot of flow cytometry gating strategy used to analyse apoptotic cells. Gates were set based on single color staining of AnnexinV and PI. Populations were distinguished as followed: early apoptotic (AnnexinV+/PI−), late apoptotic (AnnexinV+/PI+), alive (AnnexinV−/PI−) and dead (AnnexinV−/PI+) cells. Apoptosis data after **(B)** 3 h (N = 7), **(C)** 6 h (N = 5) and **(D)** 24 h (N = 6) of vehicle or MPO treatment. Statistical differences were assessed by using one-way ANOVA with Dunnett`s post hoc test.
* $p < 0.05$, ** $p < 0.01$.

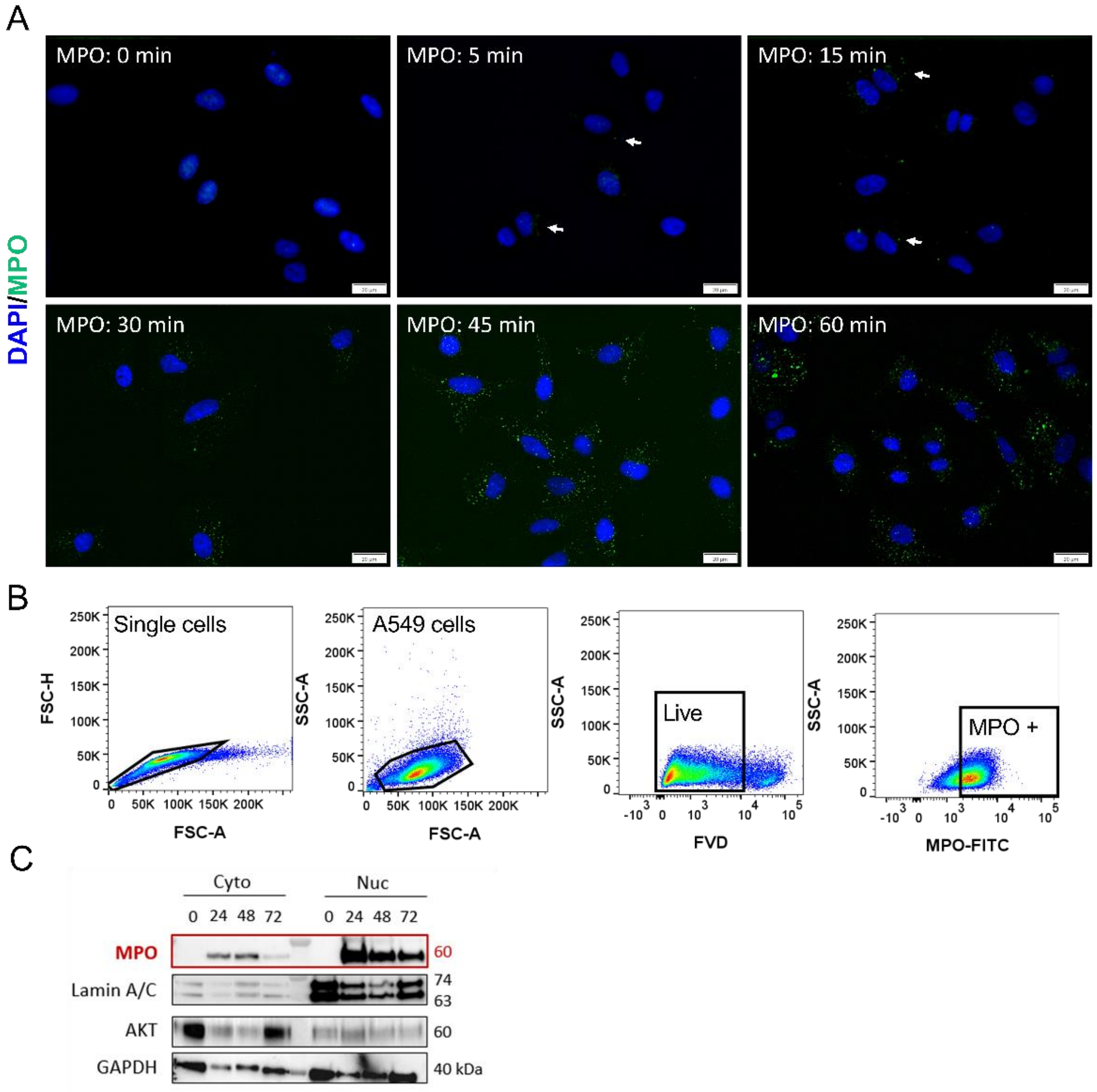

Figure S3: MPO uptake by A549 cells. (**A**) Immunofluorescence staining of A549 cells treated with 5 µg/mL MPO for indicated time (scale bar = 20 µm). (**B**) Representative scatter plot of flow cytometry gating strategy used to analyse MPO+ cells. (**C**) Representative western blot showing cytoplasmic (= Cyto) and nuclear (= Nuc) fractions of A549 cells treated with 5 µg/mL of MPO for 24, 48, and 72 h.

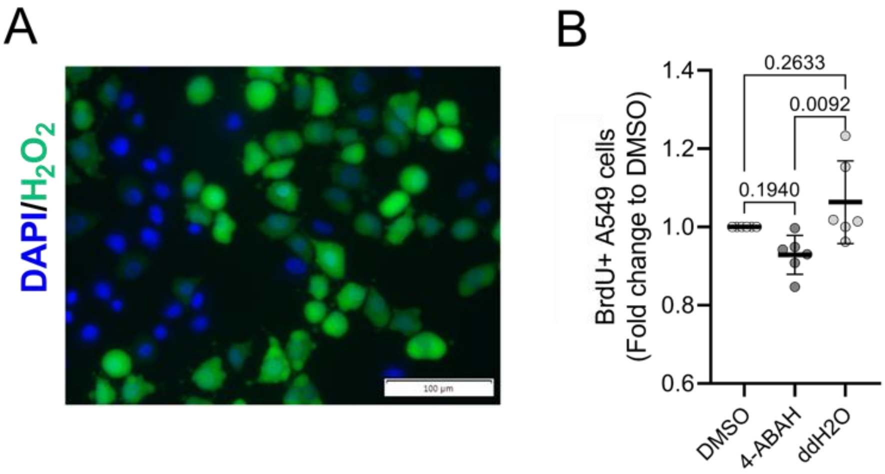

Figure S4: Intracellular MPO activity. (**A**) Hydrogen peroxide (H_2O_2) production in A549 cells under basal conditions assessed with the Intracellular Hydrogen Peroxide assay kit. (**B**) Proliferation of A549 cells assessed with the BrdU assay after 48 h of incubation (N = 6). Data indicate mean values ± SD from 6 independent experiments. Statistical differences were assessed using one-way ANOVA with Tukey´s post hoc test for multiple comparison * $p < 0.05$, ** $p < 0.01$.
